# What US hospitals are doing to prevent common device-associated infections during the coronavirus disease 2019 (COVID-19) pandemic: Results from a national survey in the United States

**DOI:** 10.1017/ice.2023.65

**Published:** 2023-12

**Authors:** Sanjay Saint, M. Todd Greene, Sarah L. Krein, Karen E. Fowler, Kathleen A. Linder, David Ratz, Jennifer Meddings

**Affiliations:** 1 Center for Clinical Management Research, Veterans Affairs (VA) Ann Arbor Healthcare System, Ann Arbor, Michigan; 2 Department of Internal Medicine, University of Michigan Medical School, Ann Arbor, Michigan; 3 VA/UM Patient Safety Enhancement Program, Ann Arbor, Michigan; 4 Infectious Disease Section, VA Ann Arbor Healthcare System, Ann Arbor, Michigan; 5 Department of Pediatrics, University of Michigan Medical School, Ann Arbor, Michigan

## Abstract

**Objective:**

The ways that device-associated infection prevention practices changed during the coronavirus disease 2019 (COVID-19) pandemic remain unknown. We collected data mid-pandemic to assess the use of several infection prevention practices and for comparison with historical data.

**Design:**

Repeated cross-sectional survey.

**Setting:**

US acute-care hospitals.

**Participants:**

Infection preventionists.

**Methods:**

We surveyed infection preventionists from a national random sample of 881 US acute-care hospitals in 2021 to estimate the current use of practices to prevent catheter-associated urinary tract infection (CAUTI), central line–associated bloodstream infection (CLABSI), and ventilator-associated events (VAE). We compared the 2021 results with those from surveys occurring every 4 years since 2005.

**Results:**

The 2021 survey response rate was 47%; previous survey response rates ranged from 59% to 72%. Regular use of most practices to prevent CLABSI (chlorhexidine gluconate for site antisepsis, 99.0%, and maximum sterile barrier precautions, 98.7%) and VAE (semirecumbent positioning, 93.4%, and sedation vacation, 85.8%) continued to increase or plateaued in 2021. Conversely, use of several CAUTI prevention practices (portable bladder ultrasound scanner, 65.6%; catheter reminders or nurse-initiated discontinuation, 66.3%; and intermittent catheterization, 37.3%) was lower in 2021, with a significant decrease for some practices compared to 2017 (P ≤ .02 for all comparisons). In 2021, 42.1% of hospitals reported regular use of the newer external urinary collection devices for women.

**Conclusions:**

Although regular use of CLABSI and VAE preventive practices continued to increase (or plateaued), use of several CAUTI preventive practices decreased during the COVID-19 pandemic. Structural issues relating to care during the pandemic may have contributed to a decrease in device-associated infection prevention practices.

The coronavirus disease 2019 (COVID-19) pandemic has posed unprecedented challenges and reinforced the importance of infection prevention and patient safety. The COVID-19 pandemic also underscored the important role infection prevention programs have in ensuring high-quality care, such as preventing healthcare-associated infection (HAI). Before the pandemic, the prevalence of HAIs among hospitalized acute-care patients in the United States was estimated at 3.2%.^1^ This estimate includes several potentially preventable device-associated infections, including catheter-associated urinary tract infection (CAUTI), central line–associated bloodstream infection (CLABSI), and ventilator-associated events (VAE). Fortunately, many episodes of such infections may be preventable through evidence-based prevention practices.^2^

For at least the past 20 years, numerous agencies and professional organizations have published recommendations regarding the use of key HAI prevention practices.^3–9^ Likewise, every 4 years since 2005, our team has conducted a national survey of infection preventionists to examine the diffusion, adoption, and implementation of these key practices by hospitals across the United States. Despite the published guideline recommendations, we found substantial variability in the adoption and regular use of infection prevention practices.^10^

We conducted our most recent survey during the COVID-19 pandemic, when many infection prevention programs took on additional responsibilities. In some cases, this change may have led to a reduced focus on HAIs. We were particularly interested in the impact of the COVID-19 pandemic on the current use of device-associated infection prevention practices by acute-care hospitals. We compared our results with the findings from similar surveys in the years leading up to the pandemic.^11–14^ We also assessed the extent to which some newer practices, such as external urinary collection devices in female patients, were being used.

## Methods

### Study design and data collection

This cross-sectional survey is part of an ongoing project in which, every 4 years, we ask infection preventionists across the United States what practices their hospitals are using to prevent common HAIs.^11–14^ For the first wave in 2005, a national random sample was selected from all nonfederal, general medical, and surgical hospitals with an intensive care unit and at least 50 hospital beds using the 2003 American Hospital Association (AHA) database. This same sample was used for the 2009 and 2013 surveys. In 2017, we selected a new random sample of 900 hospitals from all nonfederal, general medical, and surgical hospitals with an intensive care unit based on data from the 2013 AHA annual survey. Unlike prior years, hospitals of all bed sizes, including those with <50 beds, were included in this new random sample. In this 2021 iteration of the survey, we used the same sample as in 2017. Before the survey mailing, an Internet search was conducted to identify and remove any hospitals that had closed or were ineligible, resulting in 881 hospitals included in the 2021 survey.

The survey followed a modified Dillman approach.^15^ A presurvey letter was sent to the infection control coordinator at all hospitals, notifying them to expect the survey mailing in the next week. The initial surveys were mailed in mid-April 2021 and included $10 as an incentive to complete the survey. Two weeks after the initial mailing, a reminder letter was sent to all nonrespondents. Additional reminder surveys were mailed to nonrespondents ∼1, 2, and 3 months after the initial mailing. Respondents were given the option of completing the survey on paper and returning in a postage-paid envelope or completing the survey electronically using REDCap electronic data capture tools^16^ hosted at the University of Michigan. At hospitals that employed >1 infection preventionist, we asked that the lead infection preventionist serve as the primary respondent, although we encouraged consulting with others as needed to complete the questionnaire. This study received an exemption from the local institutional review board.

### Study measures

The current and prior surveys included questions about how often hospitals use various practices to prevent CAUTI, CLABSI, and VAE. The surveys assessed (1) practices that are generally recommended; (2) special approaches when infection rates are uncontrolled; (3) practices that are not recommended for routine use; and (4) newer prevention approaches.^6,7,17^ The frequency of use for each practice was assessed on a 5-point Likert scale (1 = “never use” through 5 = “always use”). Binary variables for each practice were generated with regular use defined as a rating of 4 (almost always) or 5 (always) coded as 1 and 0 otherwise. All surveys also asked questions about general hospital characteristics and characteristics of the infection control and prevention program. Our survey instrument is provided in the Supplementary Appendix 1 (online).

### Statistical analysis

Descriptive statistics, N (%) for categorical variables, and mean (±SD) for continuous variables were calculated for hospital characteristics and regular use of specific CAUTI, CLABSI, and VAE prevention practices. To investigate cross-sectional changes in the regular use of device-associated infection prevention practices, we used Poisson regression with robust standard errors. In all models, the 2017 wave was used as the reference wave to look at changes over time before the pandemic (2005–2013 vs 2017) as well as changes during the pandemic (2021 vs 2017). Incidence rate ratios (IRRs) and 95% confidence intervals (CIs) are presented for each infection prevention practice by survey wave as applicable. IRR values <1 indicate lower use of the respective practice, and IRR values >1 indicate greater use (relative to 2017). A *P* value <.05 was considered statistically significant. SAS version 9.4 software (SAS Institute, Cary, NC) was used for all analyses.

## Results

The 2021 survey response rate was 47% (415 of 881). Of 415 surveys, 306 (74%) were completed on paper, with the remainder completed electronically. Hospital characteristics are provided in Table 1. The average bed size of responding hospitals was 214 beds, and 34% of hospitals were affiliated with a medical school. Only 64% of hospitals reported receiving strong to very strong support for the infection control program from hospital leadership.

**Table 1.** Select Hospital Characteristics in 2021 (n = 415)

Characteristic	Mean (SD) or No (%)
Total number of adult acute care beds, mean (SD)	214.2 (218.2)
Total number of adult intensive care unit beds, mean (SD)	24.0 (32.6)
Percent of rooms that are private, mean (SD)	80.3 (29.6)
Hospital affiliated with a medical school	141 (34.2)
Strong/very strong overall support of infection prevention and control program from hospital leadership	266 (64.4)
Presence of a hospital epidemiologist	161 (40.4)
Lead infection preventionist board certified in infection prevention	276 (68.7)

SD, standard deviation.

### Practices to prevent CAUTI in 2021

The most regularly used practice for CAUTI prevention was aseptic catheter insertion and maintenance (89.3%), followed by portable bladder ultrasound scanner (65.6%) and nurse-initiated catheter discontinuation (52.4%). More hospitals reported regular use of external catheters in female patients (42.1%) than in male patients (36.2%). Almost all hospitals (98.8%) reported having an established surveillance system for monitoring urinary tract infection rates. In total, 78.7% of hospitals indicated that it was very to extremely important to hospital leadership to prevent urinary tract infections.

### Practices to prevent CLABSI in 2021

Nearly all responding hospitals reported regularly using 2 key recommended practices: maximum sterile-barrier precautions during central line insertion, and chlorhexidine gluconate for insertion-site antisepsis. The percentage of hospitals with an established surveillance system for monitoring CLABSIs was 97.2%. In total, 81.9% of hospitals indicated that it was very to extremely important to hospital leadership to prevent central venous catheter–related infections.

### Practices to prevent VAE in 2021

The practice regularly used by most hospitals (93.4%) to prevent VAE was semirecumbent positioning of the patient, followed by sedation vacation (85.8%). Most hospitals (88.8%) had an established surveillance system for monitoring VAE rates. In total, 62.3% of hospitals indicated that it was very to extremely important to hospital leadership to prevent VAE.

### Cross-sectional comparisons of CAUTI, CLABSI, and VAE practices: 2005 to 2021

The percentages of hospitals regularly using several practices to prevent CAUTI, CLABSI, and VAE between 2005 and 2021 are listed in Table 2, along with results from the Poisson regressions examining cross-sectional differences in the regular use of these practices relative to the 2017 wave. Additional survey findings related to CAUTI, CLABSI, and VAE prevention between 2005 and 2021 are presented in Supplementary Appendix 2 (online).

**Table 2.** Cross-Sectional Comparison of Practices to Prevent CAUTI, CLABSI, and VAE (2005–2021)

Prevention Practice	Wave	Hospitals Reporting Regular Use, %^a^	IRR^b^	95% CI		*P* Value
**CAUTI practice**						
Portable bladder ultrasound scanner for determining post-void residual	2005	29.6	0.41	0.34	0.48	<.001
	2009	38.8	0.53	0.46	0.61	<.001
	2013	57.2	0.78	0.71	0.87	<.001
	2017	73.1	Ref			
	2021	65.6	0.90	0.82	0.98	.02
Catheter reminder or stop order	2005	9.1	0.15	0.11	0.21	<.001
	2009	18.9	0.31	0.25	0.39	<.001
	2013	54.1	0.89	0.79	1.00	.048
	2017	60.7	Ref			
	2021	47.0	0.77	0.68	0.88	<.001
Nurse-initiated catheter discontinuation	2009	11.4	0.19	0.14	0.26	<.001
	2013	38.6	0.66	0.57	0.76	<.001
	2017	58.8	Ref			
	2021	52.4	0.89	0.79	1.00	.06
Catheter reminder or stop order OR nurse-initiated catheter discontinuation	2009	23.2	0.31	0.26	0.37	<.001
	2013	63.0	0.84	0.77	0.92	<.001
	2017	75.1	Ref			
	2021	66.3	0.88	0.81	0.96	.005
Silver-alloy Foley catheters	2005	32.4	1.22	0.99	1.49	.06
	2009	47.1	1.77	1.48	2.12	<.001
	2013	33.1	1.24	1.02	1.52	.03
	2017	26.6	Ref			
	2021	20.9	0.79	0.62	1.00	.05
External catheters in men (ie, condom catheters)	2005	13.1	0.51	0.38	0.69	<.001
	2009	10.6	0.41	0.30	0.57	<.001
	2013	13.8	0.54	0.40	0.72	<.001
	2017	25.7	Ref			
	2021	36.2	1.41	1.15	1.71	<.001
Aseptic technique during indwelling urethral catheter insertion and maintenance	2009	92.5	1.02	0.98	1.07	.24
	2013	93.2	1.03	0.99	1.07	.10
	2017	90.3	Ref			
	2021	89.3	0.99	0.95	1.03	.65
Intermittent catheterization	2009	22.1	0.46	0.38	0.57	<.001
	2013	31.3	0.66	0.55	0.78	<.001
	2017	47.5	Ref			
	2021	37.3	0.79	0.67	0.92	.003
**CLABSI practice**						
Maximum sterile barrier precautions during central catheter insertion	2005	71.6	0.74	0.69	0.78	<.001
	2009	91.0	0.93	0.90	0.97	<.001
	2013	97.9	1.00	0.98	1.02	.72
	2017	97.5	Ref			
	2021	98.7	1.01	0.01	1.03	.16
Alcohol containing chlorhexidine gluconate for antisepsis of the insertion site	2005	73.3	0.74	0.69	0.78	<.001
	2009	95.3	0.96	0.94	0.98	<.001
	2013	98.9	0.99	0.98	1.01	.25
	2017	99.6	Ref			
	2021	99.0	0.99	0.98	1.01	.27
Antimicrobial catheters (ie, chlorhexidine-silver sulfadiazine, minocycline-rifampin)	2005	35.0	0.86	0.72	1.02	.08
	2009	29.6	0.73	0.60	0.88	<.001
	2013	33.2	0.82	0.68	0.98	.03
	2017	40.7	Ref			
	2021	47.3	1.16	1.00	1.35	.049
Antimicrobial dressing with chlorhexidine (BIOPATCH® disk or Tegaderm^TM^ CHG)	2005	25.9	0.29	0.25	0.35	<.001
	2009	56.8	0.64	0.58	0.70	<.001
	2013	78.7	0.88	0.83	0.94	<.001
	2017	89.0	Ref			
	2021	91.5	1.03	0.99	1.07	.20
**VAE practice**						
Semirecumbent positioning of the patient (head of bed elevated ≥30°)	2005	83.1	0.85	0.81	0.89	<.001
	2009	95.6	0.97	0.95	1.00	.03
	2013	98.7	1.01	0.99	1.02	.50
	2017	98.2	Ref			
	2021	93.4	0.95	0.92	0.98	.001
Antimicrobial mouth rinse (eg, Peridex^TM^)	2005	41.5	0.50	0.44	0.57	<.001
	2009	59.3	0.71	0.65	0.78	<.001
	2013	80.5	0.97	0.91	1.03	.31
	2017	83.2	Ref			
	2021	79.4	0.95	0.89	1.02	.16
Subglottic secretion drainage (via a special endotracheal tube)	2005	22.7	0.40	0.32	0.49	<.001
	2009	42.7	0.75	0.65	0.86	<.001
	2013	56.2	0.98	0.87	1.11	.77
	2017	57.2	Ref			
	2021	53.8	0.94	0.83	1.07	.34
Topical and/or systemic antibiotics for selective digestive tract decontamination	2005	13.2	0.54	0.39	0.73	<.001
	2009	24.2	0.98	0.77	1.25	.88
	2013	25.6	1.04	0.82	1.31	.76
	2017	24.7	Ref			
	2021	22.3	0.90	0.70	1.16	.43
Silver-coated endotracheal tube	2009	5.1	0.66	0.39	1.14	.13
	2013	5.5	0.71	0.42	1.20	.20
	2017	7.7	Ref			
	2021	10.5	1.36	0.88	2.12	.17
“Sedation vacation” (ie, regular interruption of sedation)	2009	79.6	0.94	0.88	1.00	.04
	2013	85.2	1.00	0.95	1.06	.93
	2017	85.0	Ref			
	2021	85.8	1.01	0.95	1.07	.75

CAUTI, catheter-associated urinary tract infection; CLABSI, central line–associated bloodstream infection; VAE, ventilator-associated events; IRR, incidence rate ratio; CI, confidence interval; CHG, chlorhexidine gluconate.

a Presented values represent the percentage of hospitals reporting regular use (defined as responses of almost always (4) or always (5) from 5-point Likert response questions) of the prevention practice.

b All model results based on Poisson regression with robust standard errors and 2017 used as the referent group. IRR values <1 indicate lower rates of use and values >1 indicate higher rates of use, relative to 2017.

For CAUTI, the regular use of portable bladder ultrasound scanners, urinary catheter reminders or stop orders or nurse discontinuation, and intermittent catheterization was significantly lower in 2005, 2009, and 2013 compared to 2017. Although the regular use of these practices increased every 4 years between 2005 and 2017, use of these practices decreased from 2017 to 2021: portable bladder ultrasound scanners (IRR, 0.90; 95% CI, 0.82–0.98; *P* = .02), urinary catheter reminders or stop orders or nurse discontinuation (IRR, 0.88; 95% CI, 0.81–0.96; *P* = .005), and intermittent catheterization (IRR, 0.79; 95% CI, 0.67–0.92; *P* = .003). External catheters in men (eg, condom catheters, glans-adherent devices) were used less frequently in years prior to 2017, but use increased in 2021 (IRR, 1.41; 95% CI, 1.15–1.71; *P* < .001). For CLABSI, regular use of antimicrobial dressings with chlorhexidine was significantly lower in 2005, 2009, and 2013 compared to 2017 but stabilized between 2017 and 2021 (IRR, 1.03; 95% CI, 0.99–1.07; *P* = .20). Regular use of maximum sterile barrier precautions and chlorhexidine gluconate for insertion-site antisepsis has been nearly universal and stable since 2013. For VAE, compared to 2017, use of antimicrobial mouth rinse and subglottic secretion drainage was also lower in 2005 and 2009 but has remained stable since 2013. Additionally, although use of semirecumbent positioning was significantly lower in 2005 (vs 2017), this practice has been nearly universal since 2009.

## Discussion

We conducted a national survey to ascertain what US hospitals were doing to prevent 3 common device-associated infections in 2021, and we compared these findings with previous surveys dating back to 2005. The timing of the most recent survey allowed us to assess whether progress in the adoption of infection prevention practices continued during the COVID-19 pandemic. We report 3 main findings. First, the use of certain practices to prevent CLABSI and VAE continued to increase or reached a plateau over this 16-year period. These practices include the use of maximum sterile-barrier precautions during central catheter insertion and alcohol-containing chlorhexidine gluconate or antimicrobial dressing with chlorhexidine for site antisepsis (to prevent CLABSI), and sedation vacation (to prevent VAE). Second, use of several recommended practices to prevent CAUTI has decreased significantly since 2017, after rising from 2005 to 2017. This decrease included the use of a portable bladder ultrasound scanner for determining post-void residual, urinary catheter reminders or nurse-initiated catheter discontinuation, and intermittent catheterization. Third, over 40% of hospitals reported regularly using the newer tools of external urinary catheter devices for female patients, which exceeded the percentage reporting regular use of external catheters in male patients.

Infection prevention programs played an integral role in the COVID-19 response, helping to navigate and suggest safe COVID-19–related care. However, the need to focus on preventing endemic device-associated infections did not abate during this period, and some device-associated infection rates increased during the pandemic. For example, in a 148-hospital study conducted by Baker et al^18^ between March and September 2020, rates of CLABSI were 60% higher and CAUTI were 43% higher than the levels predicted if there had been no COVID-19 cases during this period.^18^ Our most recent survey, conducted in 2021, provides an overview of what US hospitals reported doing to prevent common device-associated infections during the COVID-19 pandemic. Moreover, these data, along with data collected before the pandemic, present a unique perspective on how the use of device-associated infection prevention practices was potentially affected by the COVID-19 pandemic.

The most striking finding involved CAUTI prevention practices. In our previous surveys between 2005 and 2017, we observed a general increase in the use of recommended CAUTI preventive practices. However, the reported regular use of urinary-catheter reminders or stop orders for catheter discontinuation, nurse-initiated removal protocols, portable bladder ultrasound scanners, and intermittent catheterization all decreased significantly in 2021 (compared with 2017). Notably, these practices were all part of a previous national CAUTI collaborative that reported a 32% decrease in CAUTI rates among medical-surgical patients.^19^ The reason that use of these recommended practices has decreased is unclear, but given how overworked many of the nursing staff were during the COVID-19 pandemic, CAUTI prevention may have been considered less of a priority. Additionally, because use of intermittent straight catheters, bladder scanners, and non-catheter strategies for urinary collection require more time and visits by nurses to the bedside, these practices may have been utilized less to reduce contact with COVID patients.

One exception to the general decrease in the use of CAUTI preventive practices was a significant increase in the use of condom catheters in male patients. Such devices have data supporting their use including both a randomized trial^20^ and observational data,^21^ revealing that patients prefer them to indwelling urethral catheters. Similarly, but more surprising, was the 42% of responding hospitals reporting regular use of external catheters in female patients. These devices are a newer catheter alternative for female patients (first available in 2016) and can be used for the same clinical indications as condom catheters in male patients.^22^ Although data on the benefits and risks of female external catheters are still evolving,^23–25^ several studies have shown reductions in CAUTI or indwelling catheter use after the introduction of external catheters for female patients.^24,26,27^

For CLABSI prevention, the reported regular use of maximum sterile-barrier precautions during catheter insertion and alcohol-containing chlorhexidine gluconate for insertion-site antisepsis is nearly universal in US hospitals, likely related to the robust evidence for their use^28,29^ and their incorporation as part of CLABSI “bundles” used in large-scale collaboratives.^30,31^ Two additional practices to prevent CLABSI (ie, use of antimicrobial catheters and antimicrobial dressing with chlorhexidine) also showed increases in use between the 2017 and 2021 surveys. Antimicrobial catheters are now routinely used in nearly half of US hospitals. The use of chlorhexidine-containing antimicrobial dressings has increased since our initial survey in 2005, and they are now used in over 90% of US hospitals. Although we cannot differentiate by type (Biopatch® chlorhexidine-impregnated disks vs Tegaderm^TM^ CHG dressings), we suspect that chlorhexidine-impregnated disks have been used most. Additionally, our data suggest that newer, advanced securement devices (eg, Tegaderm™ IV Advanced and SecurAcath®) were also used in over 90% of US hospitals in 2021. This widespread use could be related to continued focus on CLABSI prevention as a measure of hospital quality, overall comfort with using chlorhexidine-containing products, and the effort to minimize life-threatening infections.

For VAE, the percentage of hospitals reporting regular use of the practices we asked about remained generally similar over time, except for semirecumbent positioning, the use of which declined significantly from ∼98% regular use in 2017 to ∼93% in 2021. Some of this decrease may be directly related to the increasing use of prone positioning in patients with acute respiratory distress syndrome, as seen with severe COVID-19.^32^ Notably, the reported use of antimicrobial mouth rinse seems to have plateaued, with nearly 80% of US hospitals reporting regular use.

Our survey did note decreased use of some device-associated infection prevention practices in 2021 compared to 2017, which could be indirectly related to COVID-19. For example, issues around reducing face-to-face patient care in the setting of COVID-19 (due to personal protective equipment shortages or personnel shortages) may have decreased the number of encounters in which patients were assessed for device removal. Indwelling catheters may have been used in lieu of intermittent straight catheterization to reduce staff burden or to reduce the number of encounters between healthcare staff and patients. Shared devices, like bladder ultrasound machines, may not have been available to patients in isolation rooms. Eliminating some of these recommended infection prevention practices may have contributed to increased national HAI rates, as noted by Baker et al,^18^ during the COVID-19 pandemic. As shown by Lastinger et al,^33^ VAE, CLABSI, and (to a modest extent) CAUTI rates continued to increase during the second year of the pandemic in 2021, potentially stemming from the different types of patients admitted to hospitals during this time, increased device use (particularly ventilators), COVID-19–related comorbidity, and increased lengths of stay.

Despite using national sampling and achieving a reasonable response rate for healthcare worker surveys, especially during a pandemic, this study had several limitations. First, our surveys relied on self-reporting by respondents. Thus, it is possible that our respondents understated or overstated the use of various practices, though we expected that the lead infection preventionist would be aware of practices used in their hospital to prevent common device-associated infections. Second, although we surveyed ∼10% of all US hospitals and employed a sampling strategy to obtain a nationally representative sample, the hospitals choosing to participate may differ from those choosing not to participate, affecting the generalizability of these results. Third, during the 16 years our survey instrument has been used, we did change the term “ventilator-associated pneumonia” to VAE; the Centers for Disease Control and Prevention transitioned to this term about a decade ago.^34^ Importantly, the 2017 and 2021 survey instruments both used VAE. Finally, although the sampling scheme used for the 2017 and 2021 waves was the same, it differed from earlier waves. Notably, the sample for the 2017 and 2021 waves was expanded to include smaller hospitals with total bed sizes of less than 50. Relatedly, our findings across 5 survey waves do not reflect pure longitudinal changes among a specific group of hospitals. Still, we are confident that, despite sampling differences, our findings are robust and nationally representative across all periods of our repeated cross-sectional survey.

Despite these limitations, our national survey provides a timely overview of the practices US hospitals were using to prevent common device-associated infections in 2021, and how the use of those practices has changed over time and in the context of the COVID-19 pandemic. The regular use of practices intended to prevent CLABSI and VAE continue to increase, with several key practices reaching levels of near universal use, whereas some practices to prevent CAUTI have decreased since 2017. Dynamic patient characteristics and structural issues relating to care during the pandemic may have contributed to a decrease in some infection prevention practices. Still, our results demonstrate the importance of hard-wiring processes and routinizing infection prevention practices to withstand future pandemics, other healthcare emergencies, or organizational disruptions or system shocks (eg, the introduction of new electronic medical record systems, financial hardships, or changes in leadership).^35^ Surveys such as ours allow clinicians, hospital epidemiologists, infection preventionists, and other key decision makers to tailor their approaches to device-associated infection prevention with the goal of reducing this common complication of hospitalization.

## Supplementary material

For supplementary material accompanying this paper visit https://doi.org/10.1017/ice.2023.65. click here to view supplementary material
