# Supplementary material for: What US hospitals are doing to prevent common device-associated infections during the coronavirus disease 2019 (COVID-19) pandemic: Results from a national survey in the United States
Source: Infect Control Hosp Epidemiol. 2023 Jun 1;44(12):1913–9. doi: 10.1017/ice.2023.65 (PMC10755151; doi:10.1017/ice.2023.65)
Supplement: Supplementary file 1 [file S0899823X2300065Xsup.zip › S0899823X2300065Xsup001.pdf]

Supplementary Appendix – Survey Instrument

Translating Healthcare-Associated Infection Prevention Research into Practice Survey 2021

MICHIGAN MEDICINE
UNIVERSITY OF MICHIGAN

The University of Michigan and
The VA Ann Arbor Healthcare System
Translating Infection Prevention Team

Every 4 years since 2005 we have conducted surveys to examine the diffusion, adoption, and implementation of key practices in the field of patient safety and healthcare-associated infection prevention by hospitals across the United States. We are pleased to report that, thanks to many of you who may have participated, we found a number of interesting results related to prevention of healthcare-associated infection. If you are interested in seeing our results, you are welcome to view our published manuscripts at the following website: https://psep.med.umich.edu/trip.html

Over the past several years, there have been many changes in healthcare-associated infection prevention. Additionally, the COVID-19 pandemic has reinforced the importance of infection prevention. We are interested in what infection prevention practices hospitals are currently using.

The entire survey should take you approximately 30-45 minutes to complete. Your answers are completely confidential and only aggregate results will be reported.

If you would prefer to complete this survey electronically, you may do so at this link:

{LINK}

Who should complete this survey:

- If there is more than one Infection Preventionist (IP) at your hospital, then the IP who supervises and/or coordinates the other IPs should complete the survey.
- If there is not an IP at your hospital, then the survey should be completed by the Hospital Epidemiologist. If there is not a Hospital Epidemiologist or an IP, then the survey should be completed by either the Chair of the Infection Control Committee, the individual responsible for quality monitoring, or the Chief of Nursing.

If you work for a healthcare system where there is more than one hospital, please respond to all of the survey questions with respect to the primary acute care hospital at which you work.

There are no right or wrong answers to the infection control practice questions – we are simply interested in the strategies that are being used for infection prevention.

By returning this survey you are agreeing to participate in this research project.

Thank you for your help!

QUESTIONS ABOUT YOUR HOSPITAL:

1. Total number of adult acute care beds at your hospital? _________

2. Total number of adult Intensive Care Unit (ICU) beds at your hospital (please include all medical, surgical, and cardiac)? _________

3. Approximately what percentage of your rooms are currently:
   a. Private (1 patient) _______ %
   b. Non-private (2 or more patients) _______ %

4. Is your hospital affiliated with a medical school?
   - ☐1 No
   - ☐2 Yes
   - ☐3 Don't know

5. At your hospital, does the highest-ranking physician (e.g., Chief Medical Officer, Chief of Staff) provide direct patient care?
   - ☐1 No
   - ☐2 Yes
   - ☐3 Don't know

6. At your hospital, does the highest-ranking nurse (e.g., Chief Nurse, Director of Patient Care Services) provide direct patient care?
   - ☐1 No
   - ☐2 Yes
   - ☐3 Don't know

QUESTIONS ABOUT YOUR INFECTION CONTROL PROGRAM:

Here are some definitions to assist you when answering the following questions:

Hospital Epidemiologist: MD, PhD, or RN with expertise in hospital epidemiology and infection control.

Infection Preventionist: Individual, other than the Hospital Epidemiologist, responsible for the management and day-to-day activities of the Infection Control Program (with or without Certification in Infection Control).

7. How would you rank the overall support (e.g., staffing, financial, and political) your infection prevention and control program receives from the hospital administrative leadership?
   - ☐1 Poor
   - ☐2 Fair
   - ☐3 Good
   - ☐4 Very Good
   - ☐5 Excellent

8. Does your hospital have a Hospital Epidemiologist?
   - ☐1 No
   - ☐2 Yes
   - ☐3 Don't know

QUESTIONS ABOUT THE INFECTION PREVENTIONISTS AT YOUR HOSPITAL:

9. How many Infection Preventionists does your Infection Control Program have?

   a. ___________ Full-time

   b. ___________ Part-time

10. Is your "Lead" Infection Preventionist board certified in infection prevention and control (e.g., CIC®)? If there is more than one Infection Preventionist, the "Lead" Infection Preventionist is the one who supervises and/or coordinates the other Infection Preventionists.
   - ☐1 No
   - ☐2 Yes
   - ☐3 Don't know

11. The following pertain only to the person completing this survey. For each of the statements below please indicate the extent of your **agreement or disagreement** by checking the appropriate box.

	**Strongly disagree**	**Disagree**	**Neither disagree nor agree**	**Agree**	**Strongly agree**
A. I feel burned out from my work.	☐1	☐2	☐3	☐4	☐5
B. I have become more callous towards people since I took this job.	☐1	☐2	☐3	☐4	☐5
C. If given the opportunity to revisit my career choice, I would choose to become an infection preventionist again.	☐1	☐2	☐3	☐4	☐5
D. Spiritual well-being is important for one's emotional well-being.	☐1	☐2	☐3	☐4	☐5
E. Religious or spiritual beliefs act as a source of comfort and strength during life's ups and downs.	☐1	☐2	☐3	☐4	☐5
F. An organized religious or spiritual community is important to me.	☐1	☐2	☐3	☐4	☐5
G. Individual self-care practices (e.g., meditation, yoga, listening to music, exercising, communing with nature) is important to me.	☐1	☐2	☐3	☐4	☐5

12. The following questions ask about your views of the hospital where you work. For each of the statements below please indicate the extent of your **agreement or disagreement** by checking the appropriate box.

	Strongly disagree	Disagree	Neither disagree nor agree	Agree	Strongly agree
A. I assert my views on important issues, even though my supervisor may disagree.	☐1	☐2	☐3	☐4	☐5
B. I personally feel comfortable speaking up when I see a physician not clean his or her hands.	☐1	☐2	☐3	☐4	☐5
C. When a medical error occurs at this hospital, employees are encouraged to discuss mistakes in order to learn how to prevent similar future errors.	☐1	☐2	☐3	☐4	☐5
D. Leadership is driving us to be a safety-centered institution.	☐1	☐2	☐3	☐4	☐5
E. I would feel safe being treated here as a patient.	☐1	☐2	☐3	☐4	☐5
F. If you make a mistake at this hospital, it is often held against you.	☐1	☐2	☐3	☐4	☐5
G. Employees at this hospital are able to bring up problems and tough issues.	☐1	☐2	☐3	☐4	☐5
H. It is safe to try something new at this hospital.	☐1	☐2	☐3	☐4	☐5
I. At this hospital, people are too busy to invest time in improvement.	☐1	☐2	☐3	☐4	☐5
J. In this hospital, employees are expected to question leadership.	☐1	☐2	☐3	☐4	☐5
K. In this hospital, authority is concentrated at the top.	☐1	☐2	☐3	☐4	☐5

IF YOU HAPPEN TO HAVE BOTH ACUTE AND LONG-TERM CARE BEDS AT YOUR HOSPITAL PLEASE ANSWER THE REMAINING SURVEY QUESTIONS ONLY FOR ADULT ACUTE CARE:

CATHETER-ASSOCIATED URINARY TRACT INFECTION:

13. The following pertain to adults in your acute care hospital who require **urinary collection and/or urinary output monitoring**.

**Practices to prevent catheter-associated urinary tract infection**	Using a scale from 1 to 5 (1 being never and 5 being always), please indicate **how frequently the practice is used** in your hospital.				
A. Portable bladder ultrasound scanner for determining post-void residual	1 Never	2	3	4	5 Always
B. Urinary catheter reminder or stop-order	1 Never	2	3	4	5 Always
C. Nurse-initiated urinary catheter discontinuation	1 Never	2	3	4	5 Always
D. Silver alloy Foley catheters	1 Never	2	3	4	5 Always
E. External catheters in men (e.g., condom catheters, glans-adherent devices)	1 Never	2	3	4	5 Always
F. External catheters in women (e.g., wicking devices such as PureWick™ and PrimaFit®)	1 Never	2	3	4	5 Always
G. Aseptic technique during indwelling urethral catheter insertion and maintenance	1 Never	2	3	4	5 Always
H. Intermittent catheterization	1 Never	2	3	4	5 Always
I. Restricted list of appropriate clinical indications to place indwelling urinary catheters	1 Never	2	3	4	5 Always

14. Does your hospital have a system for monitoring which patients have urinary catheters placed?
- ☐1 No
- ☐2 Yes, but only in some units (e.g., ICU)
- ☐3 Yes, hospital wide
- ☐4 Don't know

15. Where are the **majority** of indwelling urinary catheters placed at your hospital? (select only one)
   - ☐1 Emergency Department
   - ☐2 Operating Room
   - ☐3 Floor (including telemetry/step down)
   - ☐4 Intensive Care Unit
   - ☐5 Other, please specify: ___________________________________

16. Does your hospital conduct daily rounds to assess the ongoing necessity of indwelling urinary catheters?
   - ☐1 No
   - ☐2 Yes, but only in some units (e.g., ICU)
   - ☐3 Yes, hospital wide
   - ☐4 Don't know

17. Does your hospital routinely monitor duration and/or discontinuation of urinary catheters?
   - ☐1 No
   - ☐2 Yes, but only in some units (e.g., ICU)
   - ☐3 Yes, hospital wide
   - ☐4 Don't know

18. Does your hospital have an established surveillance system for monitoring urinary tract infection rates?
   - ☐1 No
   - ☐2 Yes, but only in some units (e.g., ICU)
   - ☐3 Yes, hospital wide
   - ☐4 Don't know

19. Does your hospital report urinary tract infection rates to direct care providers?
   - ☐1 No
   - ☐2 Yes, but only in some units (e.g., ICU)
   - ☐3 Yes, hospital wide
   - ☐4 Don't know

20. Does your hospital report urinary catheter utilization ratios to direct care providers?
   - ☐1 No
   - ☐2 Yes, but only in some units (e.g., ICU)
   - ☐3 Yes, hospital wide
   - ☐4 Don't know

21. What is your perception of how important it is to hospital leadership at your hospital to prevent urinary tract infections?
   - ☐1 Minimally important
   - ☐2 Moderately important
   - ☐3 Very important
   - ☐4 Extremely important

22. Does your hospital use any of the following strategies to reduce inappropriate urine testing (i.e., diagnostic stewardship)? (select all that apply)

- ☐1 Require indication when placing order for urine test
- ☐2 Two-step or held orders to run urine cultures in emergency room
- ☐3 Encourage appropriate urine testing using decision-support or electronic alerts (e.g., best practice alerts)
- ☐4 Rejecting contaminated urine specimens
- ☐5 Hiding entire urine culture results and releasing only at provider request
- ☐6 Selective suppression of antimicrobial susceptibility results (e.g., cascade reporting)
- ☐7 Inclusion of wording or phrasing in the urine culture results to remind clinicians not to treat asymptomatic bacteriuria
- ☐8 Reflex urine testing (i.e., only perform urine culture when urinalysis is positive)
- ☐9 Nurse-directed education/initiative to reduce urine testing
- ☐10 Remove/limit urine culture testing in order sets

23. Does your hospital perform routine urine tests (for example, urinalysis and/or urine culture) to screen for urinary tract infection at the time of hospital admission?

- ☐1 No
- ☐2 Yes
- ☐3 Don't know

CENTRAL VENOUS CATHETER-RELATED INFECTION:

24. The following pertain to adults hospitalized in your facility who require **short-term central venous catheters**.

**Practices to prevent central venous catheter-related infection**	Using a scale from 1 to 5 (1 being never and 5 being always), please indicate **how frequently the practice is used** in your hospital.
A. Maximum sterile barrier precautions (full gown, sterile gloves, full body sterile drape) during central catheter insertion	1 Never 2 3 4 5 Always
B. Alcohol-containing chlorhexidine gluconate for skin antisepsis at the insertion site	1 Never 2 3 4 5 Always
C. Advanced securement device (e.g., Tegaderm™ IV Advanced, SecurAcath®)	1 Never 2 3 4 5 Always
D. Impregnated or antiseptic coated catheters	1 Never 2 3 4 5 Always
E. Use of cyanoacrylate glue at the exit site	1 Never 2 3 4 5 Always
F. Antimicrobial dressing with chlorhexidine (Biopatch™)	1 Never 2 3 4 5 Always
G. Restricted list of appropriate clinical indications to place central venous catheters	1 Never 2 3 4 5 Always

25. Does your hospital have an established surveillance system for monitoring central venous catheter-related infection rates?
    - ☐1 No
    - ☐2 Yes, but only in some units (e.g., ICU)
    - ☐3 Yes, hospital wide
    - ☐4 Don't know

26. Does your hospital report central venous catheter-related infection rates to direct care providers?
    - ☐1 No
    - ☐2 Yes, but only in some units (e.g., ICU)
    - ☐3 Yes, hospital wide
    - ☐4 Don't know

27. Who is responsible for inserting the **majority** of **non-peripherally inserted central venous catheters** at your hospital? (select only one)
    - ☐1 Designated Vascular Access Nurse or Nursing Team
    - ☐2 Interventional Radiologists
    - ☐3 Hospitalists
    - ☐4 Emergency Room Physicians
    - ☐5 Intensive Care Unit Physicians
    - ☐6 Respiratory Therapists
    - ☐7 Other, please specify: ____________________________________

28. Does your hospital have a process to determine the appropriateness of central venous catheters prior to placement?
    - ☐1 No
    - ☐2 Yes
    - ☐3 Don't know

29. Who is responsible for inserting the **majority** of **peripherally inserted central catheters (PICCs)** at your hospital?
    - ☐1 Designated Vascular Access Nurse or Nursing Team
    - ☐2 Interventional Radiologists
    - ☐3 Hospitalists
    - ☐4 Emergency Room Physicians
    - ☐5 Intensive Care Unit Physicians
    - ☐6 Respiratory Therapists
    - ☐7 Other, please specify: ____________________________________

30. Does your hospital have a process to determine the appropriateness of PICCs prior to placement?
    - ☐1 No
    - ☐2 Yes
    - ☐3 Don't know

31. Does your hospital use appropriateness guidelines (e.g., Michigan MAGIC) for PICC use?
    - ☐1 No
    - ☐2 Yes
    - ☐3 Don't know

32. Does your hospital conduct daily rounds to assess the ongoing necessity of PICCs?
   - ☐1 No
   - ☐2 Yes, but only in some units (e.g., ICU)
   - ☐3 Yes, hospital wide
   - ☐4 Don't know

33. What is your perception of how important it is to hospital leadership at your hospital to prevent central venous catheter-related infections?
   - ☐1 Minimally important
   - ☐2 Moderately important
   - ☐3 Very important
   - ☐4 Extremely important

VENTILATOR-ASSOCIATED EVENTS:

34. The following questions pertain to adults in your acute care hospital who require **mechanical ventilation**.

**Practices to prevent ventilator-associated events**	Using a scale from 1 to 5 (1 being never and 5 being always), please indicate **how frequently the practice is used** in your hospital.
A. Semi-recumbent positioning of the patient (head of bed elevated 30 degrees or more)	1 2 3 4 5<br>Never Always
B. Antimicrobial mouth rinse (e.g., Peridex®)	1 2 3 4 5<br>Never Always
C. Subglottic secretion drainage (via a special endotracheal tube)	1 2 3 4 5<br>Never Always
D. Topical and/or systemic antibiotics for selective digestive tract decontamination	1 2 3 4 5<br>Never Always
E. Silver-coated endotracheal tube	1 2 3 4 5<br>Never Always
F. "Sedation vacation" (e.g., regular interruption of sedation)	1 2 3 4 5<br>Never Always

35. Does your hospital encourage early mobilization of ventilated patients as a strategy to prevent ventilator-associated events?
   - ☐1 No
   - ☐2 Yes, but only in some units (e.g., ICU)
   - ☐3 Yes, hospital wide
   - ☐4 Don't know

36. Does your hospital have an established surveillance system for monitoring ventilator-associated event rates?
   - ☐1 No
   - ☐2 Yes, but only in some units (e.g., ICU)
   - ☐3 Yes, hospital wide
   - ☐4 Don't know

37. Does your hospital report ventilator-associated event rates to direct care providers?
- ☐1 No
- ☐2 Yes, but only in some units (e.g., ICU)
- ☐3 Yes, hospital wide
- ☐4 Don't know

38. What is your perception of how important it is to hospital leadership at your hospital to prevent ventilator-associated events?
- ☐1 Minimally important
- ☐2 Moderately important
- ☐3 Very important
- ☐4 Extremely important

CLOSTRIDIOIDES DIFFICILE INFECTION:

39. Does your hospital use supplemental no-touch disinfection devices for rooms used to care for patients with *C. difficile* infection (e.g., ultraviolet germicidal irradiation, hydrogen peroxide vapor)?
- ☐1 No
- ☐2 Yes, but only in some units (e.g., ICU)
- ☐3 Yes, hospital wide
- ☐4 Don't know

40. Does your hospital use real-time methods to assess thoroughness of cleaning and disinfection of environmental surfaces in patient rooms (e.g., fluorescent marker, adenosine triphosphate (ATP) testing)?
- ☐1 No
- ☐2 Yes, but only in some units (e.g., ICU)
- ☐3 Yes, hospital wide
- ☐4 Don't know

41. Does your hospital have a written policy to routinely test for *C. difficile* when patients have diarrhea while on antibiotics or within several months of taking them?
- ☐1 No
- ☐2 Yes, but only in some units (e.g., ICU)
- ☐3 Yes, hospital wide
- ☐4 Don't know

42. Does your hospital have an established surveillance system for monitoring *C. difficile* infection rates?
- ☐1 No
- ☐2 Yes, but only in some units (e.g., ICU)
- ☐3 Yes, hospital wide
- ☐4 Don't know

43. Are clinicians at your hospital educated as to when to order *C. difficile* testing?
- ☐1 No
- ☐2 Yes
- ☐3 Don't know

44. Which test does your laboratory primarily use to determine the presence of *C. difficile*? (select only one)

- ☐1 Antigen enzyme immunoassay
- ☐2 Toxin enzyme immunoassay
- ☐3 Polymerase chain reaction (PCR)
- ☐4 Culture/Cytotoxin assay
- ☐5 Some combination of the above

45. Does your hospital use any of the following strategies to reduce inappropriate testing for *C. difficile* (i.e., diagnostic stewardship)? (select all that apply)

- ☐1 Reject formed stool submitted for *C. difficile* testing
- ☐2 Reject testing for *C. difficile* on patients who have received laxatives within last 72 hours
- ☐3 Discourage testing for *C. difficile* on patients on laxatives using decision-support or electronic alerts (e.g., best practice alerts)
- ☐4 Use of cascade testing or hiding test results
- ☐5 Other, please specify: ____

46. Does your hospital offer Fecal Microbiota Transplant (FMT) for patients with recurrent *C. difficile* infection?

- ☐1 No
- ☐2 Yes
- ☐3 Don't know

47. Does your hospital report *C. difficile* infection rates to direct care providers?

- ☐1 No
- ☐2 Yes, but only in some units (e.g., ICU)
- ☐3 Yes, hospital wide
- ☐4 Don't know

48. What is your perception of how important it is to hospital leadership at your hospital to prevent *C. difficile* infection?

- ☐1 Minimally important
- ☐2 Moderately important
- ☐3 Very important
- ☐4 Extremely important

GENERAL INFECTION PREVENTION PRACTICES:

49. Do you feel you are under any pressure to NOT report healthcare-associated infections at your hospital?

- ☐1 No
- ☐2 Yes
- ☐3 Don't know

50. Among all patient safety issues, how important is hand hygiene at your hospital?

- ☐1 Minimally important
- ☐2 Moderately important
- ☐3 Very important
- ☐4 Extremely important

51. What was the last overall hand hygiene compliance rate reported in your hospital (between 0 and 100%)?

_______ %

52. Which surveillance method(s) does your hospital use for monitoring hand hygiene compliance (select all that apply)?
    - ☐1 Direct observation method by validated observer
    - ☐2 Calculation of hand hygiene product consumption
    - ☐3 Electronic system
    - ☐4 Self-report by staff
    - ☐5 Other, please specify: ____

53. Does your hospital have an antimicrobial stewardship program?
    - ☐1 No (If no, skip to question 57)
    - ☐2 Yes
    - ☐3 Don't know

54. If yes, please indicate who is on your antimicrobial stewardship team (select all that apply).
    - ☐1 Infection Preventionist
    - ☐2 Infectious Diseases Physician
    - ☐3 Hospitalist
    - ☐4 Other Physician
    - ☐5 Pharmacist (**with** infectious diseases training)
    - ☐6 Pharmacist (**without or unknown** infectious diseases training)
    - ☐7 Nurse
    - ☐8 Other, please specify: ____

55. Please indicate below if your antimicrobial stewardship program measures aggregate antibiotic use (e.g., defined daily dose, days of therapy) for use in any of the following settings: (select all that apply)
    - ☐1 Inpatient units (in hospital)
    - ☐2 Outpatient/ambulatory clinics associated with your hospital
    - ☐3 At the time of discharge from the hospital
    - ☐4 Emergency room(s)
    - ☐5 Urgent care facilities associated with your hospital
    - ☐6 Nursing homes, long-term care facilities, or rehabilitation facilities associated with your hospital
    - ☐7 None of the above

56. Please indicate below if your antimicrobial stewardship program has one or more interventions in place to improve appropriate antibiotic use in any of the following settings: (select <u>all</u> that apply)
   - ☐1 Inpatient units (in hospital)
   - ☐2 Outpatient/ambulatory clinics associated with your hospital
   - ☐3 At the time of discharge from the hospital
   - ☐4 Emergency room(s)
   - ☐5 Urgent care facilities associated with your hospital
   - ☐6 Nursing homes, long-term care facilities, or rehabilitation facilities associated with your hospital
   - ☐7 None of the above

57. Does your facility provide tele-stewardship (i.e., using remote monitoring to help other facilities)?
   - ☐1 No
   - ☐2 Yes
   - ☐3 Don't know

58. Does your facility receive tele-stewardship (i.e., another group helps you monitor antibiotic use)?
   - ☐1 No
   - ☐2 Yes
   - ☐3 Don't know

59. Does your hospital have any strategies in place to reduce inappropriate testing *for any of the following*? (select <u>all</u> that apply)
   - ☐1 Urine testing (including urine cultures or urinalyses)
   - ☐2 Blood cultures
   - ☐3 Respiratory cultures
   - ☐4 Respiratory panel by PCR
   - ☐5 Stool gastrointestinal pathogen panel by PCR

60. At your hospital, contact precautions for MRSA are required for which of the following: (select only one)
   - ☐1 All MRSA colonized or infected patients and we do MRSA surveillance culturing
   - ☐2 All MRSA colonized or infected patients, but we do NOT do MRSA surveillance culturing
   - ☐3 MRSA infected patients only
   - ☐4 MRSA colonized or infected patients under special circumstances (e.g., open wounds)
   - ☐5 Not required

61. At your hospital, contact precautions for vancomycin-resistant enterococci (VRE) are required for which of the following: (select only one)
   - ☐1 All VRE colonized or infected patients and we do VRE surveillance culturing
   - ☐2 All VRE colonized or infected patients, but we do NOT do VRE surveillance culturing
   - ☐3 VRE infected patients only
   - ☐4 VRE colonized or infected patients under special circumstances (e.g., open wounds)
   - ☐5 Not required

62. At your hospital, contact precautions for carbapenem-resistant Enterobacteriaceae (CRE) are required for which of the following: (select only one)

- ☐1 All CRE colonized or infected patients and we do CRE surveillance culturing
- ☐2 All CRE colonized or infected patients, but we do NOT do CRE surveillance culturing
- ☐3 CRE infected patients only
- ☐4 CRE colonized or infected patients under special circumstances (e.g., open wounds)
- ☐5 Not required

63. The following pertain to adults hospitalized in your acute care facility.

**Infection prevention practice**	Using a scale from 1 to 5 (1 being never and 5 being always), please indicate **how frequently the practice is used** in your hospital
A. Decolonization of the nose and skin in patients colonized with methicillin-resistant *Staphylococcus aureus* (MRSA) prior to a surgical procedure	1 Never 2 3 4 5 Always
B. Chlorhexidine gluconate for daily bathing of ICU patients	1 Never 2 3 4 5 Always
C. Chlorhexidine gluconate for daily bathing of non-ICU patients	1 Never 2 3 4 5 Always

64. The following questions pertain to annual influenza vaccination of healthcare workers at your hospital.

a. Does your hospital mandate healthcare workers to receive annual influenza vaccination? (select only one)

- ☐1 No hospital mandates regarding influenza vaccination
- ☐2 Healthcare workers are encouraged to get vaccination, but it is not mandated
- ☐3 There is a mandate requiring healthcare workers to get vaccination, with the option of opting out for allowable reasons
- ☐4 Don't know

b. Which of the following are allowable reasons for healthcare workers to opt out or decline vaccination at your hospital? (select all that apply)

- ☐1 Medical contraindication
- ☐2 Religious reasons
- ☐3 No reason required
- ☐4 Other, please specify: ____
- ☐5 Not applicable / annual influenza vaccination not mandated

c. Are those who do not receive annual influenza vaccination required to wear a mask when providing patient care during the flu season?

- ☐1 No
- ☐2 Yes
- ☐3 Don't know

d. Are there any penalties for healthcare workers who are non-compliant with your facility's policy on influenza vaccination?

☐1 No
☐2 Yes, please specify: ____
☐3 Don't know

COVID-19: The following questions pertain to your hospital's experiences during the COVID-19 pandemic **at any point from March 2020 until the present**.

65. In your opinion, how effective has your hospital's pandemic response plan been in addressing COVID-19?

☐1 Minimally effective
☐2 Moderately effective
☐3 Very effective
☐4 Extremely effective
☐5 Not applicable, our hospital did not have a pandemic response plan

66. Which organization has your facility relied on the most for information about COVID-19? (select only one)

☐1 Centers for Disease Control & Prevention (CDC)
☐2 World Health Organization (WHO)
☐3 Centers for Medicare & Medicaid Services (CMS)
☐4 Infectious Diseases Society of America (IDSA)
☐5 Association for Professionals in Infection Prevention & Epidemiology (APIC)
☐6 State and/or local health department
☐7 Local hospital/healthcare organization
☐8 Other, please specify: ____

67. Has your hospital designated areas to care for COVID-19 patients that are separated from non-COVID patients?

☐1 No
☐2 Yes, but only in some units (e.g., ICU)
☐3 Yes, hospital wide
☐4 Don't know

68. Has your hospital opened new units to care for COVID-19 patients?

☐1 No
☐2 Yes
☐3 Don't know

69. Has your hospital experienced staff shortages due to absences and/or illness during the COVID-19 pandemic?

☐1 No
☐2 Yes
☐3 Don't know

70. Has your hospital experienced an increased loss of staff (e.g., resignations) in the midst of COVID-19?
- ☐1 No
- ☐2 Yes
- ☐3 Don't know

71. Has your hospital experienced a shortage of any supplies during the COVID-19 pandemic? (select all that apply)
- ☐1 N95 masks
- ☐2 Powered air-purifying respirators (PAPRs)
- ☐3 Alcohol-based hand sanitizer
- ☐4 Gowns
- ☐5 Gloves
- ☐6 Surgical masks
- ☐7 Full face shields
- ☐8 Eye shields/goggles
- ☐9 Disinfectant wipes
- ☐10 Other, please specify: ________________
- ☐11 No supply shortages experienced

72. Has your hospital experienced financial hardship resulting from the COVID-19 pandemic?
- ☐1 No financial hardship
- ☐2 Mild financial hardship
- ☐3 Moderate financial hardship
- ☐4 Extreme financial hardship
- ☐5 Don't know

73. Please indicate how much you agree or disagree with the following statement. "I feel safe carrying out my work role during the COVID-19 pandemic?"
- ☐1 Strongly disagree
- ☐2 Disagree
- ☐3 Neither disagree nor agree
- ☐4 Agree
- ☐5 Strongly agree

74. How confident are you that a COVID-19 vaccine will be safe and effective?
- ☐1 Not confident at all
- ☐2 Only slightly confident
- ☐3 Somewhat confident
- ☐4 Moderately confident
- ☐5 Very confident

75. If your employer does not require it, would you or have you voluntarily vaccinated yourself against COVID-19?
- ☐1 No
- ☐2 Yes
- ☐3 Don't know/Prefer not to answer

76. In your opinion, how effective has your hospital's COVID-19 vaccination plan been in vaccinating staff?

- ☐1 Minimally effective
- ☐2 Moderately effective
- ☐3 Very effective
- ☐4 Extremely effective
- ☐5 Not applicable, our hospital does not have a COVID-19 vaccination plan for staff

FINAL QUESTIONS: The following pertain only to the person completing this survey.

77. What is your position in the hospital?

- ☐1 Infection Preventionist
- ☐2 Nursing Management
- ☐3 Hospital Epidemiologist
- ☐4 Other, please specify: ____

78. How long have you had this position? ______ years _______ months

79. How long have you worked at this hospital? ______ years _______ months

Congratulations! You are finished. Thank you for completing this survey!

Please provide any additional comments below:
