# Supplementary material for: What US hospitals are doing to prevent common device-associated infections during the coronavirus disease 2019 (COVID-19) pandemic: Results from a national survey in the United States
Source: Infect Control Hosp Epidemiol. 2023 Jun 1;44(12):1913–9. doi: 10.1017/ice.2023.65 (PMC10755151; doi:10.1017/ice.2023.65)
Supplement: Supplementary file 1 [file S0899823X2300065Xsup.zip › S0899823X2300065Xsup002.pdf]

Supplementary Appendix. Use of Additional CAUTI, CLABSI and VAE prevention practices and related findings, 2005-2021[a]

Prevention Practice	2005	2009	2013	2017	2021
**CAUTI**					
External catheters in women	-	-	-	-	42.1%
Established surveillance system for monitoring urinary tract infection rates	69.4%	79.7%	97.6%	98.1%	98.8%
Hospital has system for monitoring which patients have urinary catheters	44.1%	57.3%	84.5%	94.8%	92.8%
Hospital routinely monitors duration or urinary catheters	25.9%	39.4%	78.6%	88.4%	85.9%
Hospital reports urinary tract infection rates to direct care providers	60.5%	69.4%	88.0%	90.1%	87.1%
Hospital reports urinary catheter utilization ratios to direct care providers	-	-	-	69.8%	65.0%
Very/extremely important to hospital leadership to prevent urinary tract infections	-	58.5%	74.7%	83.8%	78.7%
**CLABSI**					
Advanced securement device	-	-	-	-	91.1%
Use of cyanoacrylate glue at the exit site	-	-	-	-	5.0%
Established surveillance system for monitoring CLABSI rates	92.3%	96.7%	98.0%	98.5%	97.2%
Hospital reports central venous catheter-related infection rates to direct care providers	-	87.6%	93.4%	92.9%	89.2%
Very/extremely important to hospital leadership to prevent central venous catheter-related infections	-	80.3%	89.6%	86.8%	81.9%
**VAE**					
Established surveillance system for monitoring VAE rates	93.1%	95.6%	96.4%	93.7%	88.8%
Hospital encourages early mobilization of ventilated patients	-	-	74.7%	74.5%	74.0%
Hospital reports ventilator-associated event rates to direct care providers	-	90.7%	92.1%	87.4%	78.3%
Very/extremely important to hospital leadership to prevent VAEs	-	76.5%	77.6%	73.2%	62.3%

Abbreviations: CAUTI = catheter-associated urinary tract infection, CLABSI = central line-associated bloodstream infection, VAE = ventilator-associated event.

[a] Presented values represent the percentage of hospitals reporting regular use (defined as responses of almost always (4) or always (5) from 5-point Likert response questions) of the prevention practice, unless otherwise indicated.
